# Case Report: Novel JAG1 gene mutations in two infants with alagille syndrome characterized by cholestasis

**DOI:** 10.3389/fped.2022.1017647

**Published:** 2022-10-20

**Authors:** Yijiang Han, Kun Zhu, Hao Wu, Baohai Chen, Shuqi Hu, Dengming Lai, Jinfa Tou

**Affiliations:** ^1^ Department of Neonatal Surgery, The Children's Hospital, Zhejiang University School of Medicine, National Clinical Research Center for Child Health, Hangzhou, China; ^2^ Department of Pathology, The Children's Hospital, Zhejiang University School of Medicine, National Clinical Research Center for Child Health, Hangzhou, China; ^3^ The Children's Hospital, Zhejiang University School of Medicine, National Clinical Research Center for Child Health, Hangzhou, China; ^4^ Department of Information Center, The Children's Hospital, Zhejiang University School of Medicine, National Clinical Research Center for Child Health, Hangzhou, China

**Keywords:** infant, cholestasis, diagnosis, biliary atresia, MMP-7, alagille syndrome, JAG1, gene mutation

## Abstract

**Background:**

Infants with Alagille syndrome (ALGS) need to be promptly differentiated from biliary atresia (BA) at an early stage. ALGS is an autosomal, dominant, multisystem disorder with variable phenotypic penetrance caused by heterozygous mutations in JAG1 or NOTCH2, which encode the Notch signaling pathway.

**Case presentation:**

We report two cases, both with cholestatic jaundice as the main manifestation, in which BA was excluded and finally diagnosed as ALGS based on characteristic facial features, serological tests, imaging, laparoscopic cholangiography, pathology and genetic findings. Both cases are novel mutant genes on chromosome 20 that have not been reported in the literature. The mutation in patient 1 was a novel heterozygous nonsense mutation (NM_000214 exon20, c.2419G > T, *p*.E807Ter), which was a spontaneous mutation. Followed up to 1 year and 6 months, the symptoms resolved with ursodeoxycholic acid and cholestyramine, and the jaundice has now subsided. Patient 2 was a novel heterozygous frameshift mutation (NM_000214 exon19, c.2367–2368dupTC, *p*.P790Lfs*31), which was inherited from his mother. This patient was followed up to 9 months and is currently awaiting liver transplantation.

**Conclusion:**

Both cholestatic infants reported combined to exclude BA, avoid Kasai portoenterostomy (KPE), and definitively diagnose ALGS. Broadening the spectrum of JAG1 gene mutations.

## Introduction

Cholestatic jaundice is one of the major liver diseases in infants. Biliary atresia (BA) presents clinically in the first weeks of life with jaundice and acholic pale stools, the prototypical clinical features of an obstructive-type jaundice (1). Early diagnosis of BA and timely Kasai portoenterostomy (KPE) are correlated with favorable prognoses (2). Alagille syndrome (ALGS) is a genetic, multi-organ disorder of varying severity. The first clinical description of ALGS was proposed by French hepatologist Daniel Alagille, who reported 30 patients with intrahepatic bile duct dysplasia based on clinical observations of the liver (cholestasis, bile duct paucity), heart, eyes, skeletal system and characteristic facies. 50% of these patients appeared to have easily identifiable extrahepatic clinical features (3). With an incidence of 1:30,000 to 1:50,000 births, autosomal dominant ALGS is the result of Notch signaling dysfunction caused by mutations in genes (primarily in JAG1 and NOTCH2) (4). Haploinsufficiency caused by truncation or early transcriptional termination of JAG1 accounts for 83% of mutations seen in patients with ALGS. NOTCH2 mutations are rarely specified as the sole cause of ALGS and occur in less than 3% of patients according to a recent analysis (5).

ALGS is caused by mutations in the JAG1 or NOTCH2 genes, either of which produces single-pass, transmembrane proteins involved in the Notch signaling pathway. The NOTCH2 gene encodes a Notch protein, and JAG1 encodes the cell surface protein Jagged1, which is a ligand for the Notch receptor (6). Jagged1 expression in the portal vein interstitium is required for bile duct formation. Notch2 signaling is important for the differentiation of hepatoblasts into biliary epithelial cells and for the survival of these cells. Thus, the typical hepatic manifestation of ALGS is the paucity of interlobular bile duct (PIBD), which is due to defects in the morphology of the bile ducts (7).

In the present article we describe two patients with ALGS requiring differential diagnosis with BA carrying novel mutations that have not been reported in the literature.

## Case description

### Patient 1

**Figure 1:**
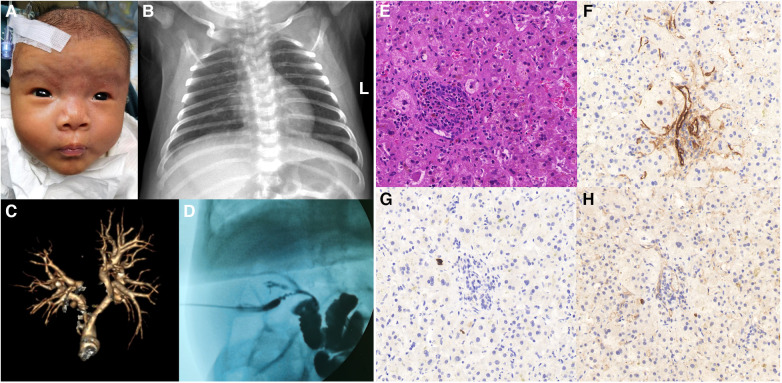
(**A**) facial features of patient 1. (**B**) Chest x-ray shows butterfly vertebra. (**C**) 3D reconstruction of the pulmonary artery shows left and right pulmonary artery stenosis. (**D**) Laparoscopic cholangiogram reveals intrahepatic bile ducts. (**E**) HE staining shows absence of bile ducts in the confluent area with inflammatory cell infiltration and swollen liver cells with visible hepatic giant cells. (**F**) Immunohistochemistry CD34 was positively expressed in the endothelial cells of interlobular veins and interlobular arteries in the confluent area. (**G**) Immunohistochemistry CK19 shows absence of bile ducts in the confluent region. (**H**) Immunohistochemistry CK7 shows absence of bile ducts in the confluent region.

The proband is a full-term baby girl who was admitted with cholestasis at 1 month and 16 days of age due to a yellowish scleral discoloration found for more than 1 month. The child also had a combination of light-colored stools and even a white clay color. There was no family history of heart or liver disease. During the pregnancy her mother had regular checkups and was not taking any medications except prenatal vitamins (vitamin D, vitamin B6, folic acid) and supplements (iron and calcium). On admission, the infant was 52 cm tall and weighed 3.05 kg. Her face showed an inverted triangular face formed by a high prominent forehead and a pointed chin, deep set eyes and hypertelorism, and a straight nose with a bulbous tip (Figure 1A). The butterfly vertebra was seen on the chest x-ray after admission (Figure 1B). The hepatobiliary ultrasound showed no significant abnormalities in liver morphology and size, no significant dilatation of the intrahepatic and extrahepatic bile ducts, and no significant fibrous plaques in the bile duct travel area. The gallbladder size was 1.6 cm*0.5 cm, and the cyst wall thickness was 0.14 cm. CT angiography (CTA), 3D reconstruction suggested right and left pulmonary artery stenosis (left ID 1.8 mm, right ID 2.4 mm) (Figure 1C) and persistent left superior vena cava (PLSVC). Liver function test results were shown in Table 1. TBA was detected by liquid chromatography-tandem mass spectrometry (LC-MS/MS) at 73.496 *μ*mol/L, including 5.202 *μ*mol/L for glycochenodeoxycholic acid (GCDCA) and <0.025 *μ*mol/L for chenodeoxycholic acid (CDCA). Serum matrix metalloproteinase-7 (MMP-7) was 10.52 ng/ml. Laparoscopic biliary exploration, cholangiography and liver biopsy were performed on the 10th day of admission. Cholangiography could show intrahepatic bile ducts and rule out BA (Figure 1D). Pathology showed absence of bile ducts in the confluent area with inflammatory cell infiltration and swollen liver cells with visible hepatic giant cells (Figure 1E). Immunohistochemistry CD34 was positively expressed in the endothelial cells of interlobular veins and interlobular arteries in the confluent area (Figure 1F). CK19 and CK7 showed absence of bile ducts in the confluent area (Figures 1G,H).

**Table 1:** Liver function test values of the two patients.

	Patient 1				Patient 2			
	On admission	6 months old	12 monthsold	18 months old	On admission	3 months old	6 months old	9 months old
DBIL (*μ*mol/L) (0–5.1)	76.2	162.2	56.2	3.6	96.7	86.0	135.2	101.8
IBIL (*μ*mol/L) (1.0–20.0)	51.5	143.9	14.6	4.9	75.8	59.8	119.3	78.1
ALT (U/L) (<50)	43	410	141	108	232	490	162	178
AST (U/L) (15–60)	102	441	117	94	264	385	201	247
GGT (U/L) (8–57)	257	303	512	349	81	527	411	355
ALP (U/L) (42-362)	408	487	455	501	690	497	480	638
TBA (*μ*mol/L) (0–13.0)	110.6	291.7	142.4	88.0	249.9	169.0	343.8	266.8
TG (mmol/L) (<1.70)	1.01	2.33	1.78	1.47	2.76	1.51	2.30	3.68
TC (mmol/L) (3.00–5.70)	3.60	9.80	7.73	8.42	7.14	7.46	6.11	13.86

DBIL, direct bilirubin; IBIL, indirect bilirubin; ALT, alanine aminotransferase; AST, aspartate aminotransferase; GGT, gamma-glutamyltransferase; ALP, alkaline phosphatase; TBA, total bile acids; TG, triglyceride; TC, total cholesterol.

To verify the suspicion of ALGS, genes were tested and analyzed. Approximately 2 ml peripheral blood (EDTA anticoagulant) of the patient and her parents was collected, and genomic DNA was extracted from peripheral blood using the QIAamp DNA Mini Kit (Qiagen, Shanghai, China) using the manufacturer's instructions. After libraries preparation, a GenCap capture panel (MyGenostics Inc, Beijing, China) with 212 genes associated with Cholestasis was sequenced using DNBSEQ-T7. Raw sequencing reads were aligned to the human reference genome hg19, and variants calling were conducted by Sentieon. Variants were further annotated by ANNOVAR and associated with multiple databases, such as gnomAD, 1,000 genomes, dbSNP, EXAC, and predicted by SIFT, PolyPhen-2, MutationTaster, GERP++, and SPIDEX. The pathogenicity of variation loci also was analyzed according to ACMG (American College of Medical Genetics and Genomics) genetic variation classification criteria and guidelines (8). The identification of candidate variants was validated in the patient and her parents by Sanger sequencing.

**Figure 2:**
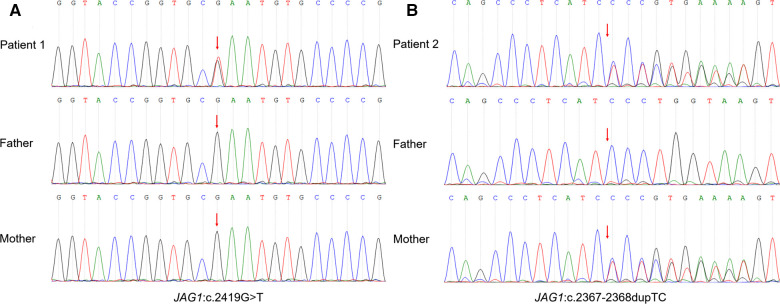
Sanger sequencing of the trios confirmed the JAG1 variant. (**A**) Patient 1: heterozygous nonsense mutation (NM_000214 exon20, c.2419G > T, *p*.E807Ter), which was a spontaneous mutation. (**B**) Patient 2: heterozygous frameshift mutation (NM_000214 exon19, c.2367–2368dupTC, *p*.P790Lfs*31), which was inherited from his mother.

A nonsense variant in JAG1 gene (NM_000214 exon20, c.2419G > T, *p*.E807Ter) was identified by cholestasis-related gene sequencing (nucleotide 2,149 in exon 20 is changed from guanine to thymine) (Figure 2A). This mutation leads to the substitution of Glutamic acid to a stop codon at codon 807 (*p*.E807Ter), which causes a truncated variant protein. Sanger sequencing showed the variant was *de novo* because her unaffected parents did not have the variant. According to the criteria proposed by the ACMG (8), this variant was classified as “pathogenic” as evidenced by PVS1 and PS2 and PM2_supporting (PVS1: the variant is a nonsense mutation that may result in loss of gene function; PS2: *de novo* (both maternity and paternity confirmed) in a patient with the disease and no family history; PM2_supporting: the variant was absent from the controls databases). No variant was detected in the patient's NOTCH2 gene. Based on the patient's facial features, congenital heart disease and cholestasis, combined with pathological and genetic findings, her phenotype was consistent with a diagnosis of ALGS.

The child was 1 year and 6 months old at the time of follow-up. After surgical exploration, she was given methylprednisolone 2 mg/kg qd orally for one week and later changed to 1 mg/kg qd orally for one week. Vitamin D supplementation and ursodeoxycholic acid 10 mg/kg bid orally also were given. High bile acid value was treated symptomatically with oral cholestyramine and occasional pruritus. The bilirubin level has now returned to normal, ALT and AST fluctuate around 100 U/L and are decreasing (Table 1), and fat-soluble vitamins are in the normal range. The yellow staining of the face and sclera has basically disappeared, and the stool color is yellow.

### Patient 2

**Figure 3:**
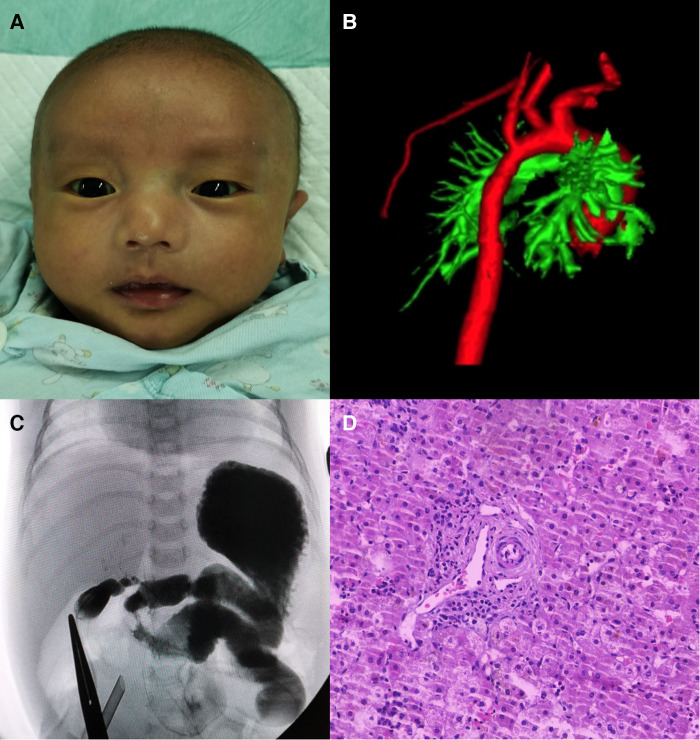
(**A**) Facial features of patient 2. (**B**) 3D reconstruction of the aorta showing stenosis of the descending aortic arch. (**C**) Laparoscopic cholangiogram shows a visualization of the intrahepatic bile ducts. (**D**) HE staining reveals interlobular arteries and veins with absent bile ducts.

The proband is a full-term male infant who was admitted to the hospital with cholestasis at 1 month and 11 days of age after being found to have yellowish skin and sclera with light colored stools for more than 1 month. There was no family history of heart or liver disease. His mother had regular checkups during her pregnancy and no history of maternal drug abuse. His mother appears to exhibit a craniofacial dysmorphism typical of ALGS. His mother had no relevant family history, no previous history of jaundice, abnormal liver function values, and no abnormalities of the heart, spine, fundus, urinary system, nervous system, etc. His height at admission was 54 cm, and the weight was 3.6 kg. He has a triangular-shaped face with a prominent forehead, hypertelorism, deep-set eyes, a pointed chin, and a bulbous nasal tip (Figure 3A). Cardiac ultrasound after admission: increased flow velocity in the aorta and descending aortic arch, increased flow velocity in the left and right pulmonary arteries, mild tricuspid regurgitation. CTA, 3D reconstruction: narrowed descending aortic arch (internal diameter about 4.2 mm) (Figure 3B). Renal ultrasound: increased resistance index in both renal arteries (left RI: 0.93, right RI:0.92). Hepatobiliary ultrasound: no significant abnormalities in liver morphology and size, no significant dilatation of intrahepatic and external bile ducts, no significant fibrous plaques in the bile duct travel area. The gallbladder size was 2.3 cm*0.6 cm. Liver function values were shown in Table 1. Serum MMP-7 was 19.89 ng/ml, GCDCA and CDCA were 36.026 *μ*mol/L and <0.025 *μ*mol/l. Laparoscopic biliary exploration, cholangiography and liver biopsy were performed on the 6th day of admission. The contrast showed tiny intrahepatic bile ducts (Figure 3C). BA was definitively ruled out. Pathology showed interlobular arteries and veins with absence of bile ducts (Figure 3D).

The genetic testing method was the same as Patient 1. A frameshift variant in JAG1 gene (NM_000214 exon19, c.2367–2368dupTC, *p*.P790Lfs*31) was identified by cholestasis-related gene sequencing (Figure 2B). This mutation introduces thirty novel amino acids after codon 789 (proline 790, as the first affected amino acid, was changed to leucine) and a premature stop codon at 821 that resulted in a truncated protein Sanger sequencing showed the variant originated from the mother. According to the criteria proposed by the ACMG (8), this variant was classified as PVS1 + PM2_supporting and was annotated as “likely pathogenic” (PVS1: the variant is a frameshift mutation that may result in loss of gene function; PM2_supporting: the variant was absent from the controls databases). The results of various tests suggest a diagnosis of ALGS.

He is now 9 months old at follow-up. After surgical exploration, he was also given methylprednisolone 2 mg/kg qd orally for one week and later changed to 1 mg/kg qd orally for one week. Vitamin D supplementation and ursodeoxycholic acid also were given orally, and cholestyramine was given orally as symptomatic treatment for high bile acid. The liver function values during follow-up were shown in Table 1. Vitamin D 0.67 ng/ml. At present, the child's skin and sclera are still visibly yellow, stools are still light yellow in color, occasionally white clay in color. He has persistent itching and is awaiting liver transplantation (LT).

## Discussion

Evaluation of neonatal cholestasis is challenging and requires timely assessment of a broad differential diagnosis. The liver is the typical organ involved in ALGS. Splenomegaly is uncommon early in the course of the disease but is present in 70% of patients of advanced age (6). Seven diagnostic signs are combined: cholestasis, congenital heart defects (most commonly pulmonary artery stenosis), skeletal anomalies (most commonly butterfly vertebrae), ocular defects (usually posterior embryotoxon), renal involvement (most commonly renal dysplasia), vascular involvement (central nervous system (CNS) vascular malformations are common), and a characteristic triangular facial appearance. If three of these signs are present, the diagnosis is confirmed (6, 9). In the presence of a family history of ALGS, the presence of the JAG1 mutation is diagnostic of ALGS even if all of the above criteria are not present. If the mutation or family history is positive, at least one major criterion is required to make the diagnosis (7). The patient's face is an important factor that raises clinical suspicion: deep-set eyes, hypertelorism, triangular shaped face and bulbous nose tip. These include one of the factors justifying genetic testing as a diagnostic approach.

ALGS in infancy is not well characterized and needs to be differentiated from BA. Histopathological features of BA liver specimens include bile ductular proliferation (BDP), portal vein infiltration, giant cells, hepatocyte swelling and fibrosis (10). The basic feature of ALGS is a reduced number of bile ducts (bile duct paucity), and almost all patients exhibit cholestatic disease (4). The presence of bile duct paucity in ALGS patients varies with age and is absent in 40% of children <6 months. Intraoperative cholangiography also may be misleading (6). KPE in ALGS may increase the extent of liver injury and may accelerate the progression of liver fibrosis and worsen the overall prognosis, and is considered a marker for future LT (11). If a diagnosis of ALGS is suspected, the initial clinical evaluation should include routine laboratory tests (liver function tests, including GGT, TG, TBA, TC, complete blood count and coagulation) and imaging tests (liver ultrasound). Depending on the clinical situation, liver tissue biopsy may or may not be performed. In infants with cholestasis suspected of BA, cholangiography and liver biopsy should be performed immediately because BA must be ruled out in time and cannot wait for the results of genetic testing. The prevailing view suggested the optimal timing for the KPE was at 45–60 days of age (1). Both cases of ALGS that we described have cholestasis as the main feature and need to be diagnosed as soon as possible as a differential with BA. Both cases may have persistent pale stools, high GGT and gallbladder dysplasia. BA was ruled out by ultrasound, liver function tests, serum MMP-7 and laparoscopic cholangiography. Serum MMP-7 can be used for early diagnosis of BA, using a cut-off value of >26.73 ng/ml, with an AUC of 0.954 in BA neonates and 0.983 in BA infants (2). ALGS was confirmed by liver tissue biopsy and genetic testing. Owing to the young age, other characteristic manifestations, such as xanthomas, have not yet been demonstrated.

ALGS is an autosomal dominant disorder with pathogenic variants occurring mainly in JAG1. Both JAG1 and NOTCH2 are single-pass transmembrane proteins with extracellular structural domains, and JAG1 (Jagged1) is the transmembrane ligand of the Notch signaling pathway (6). Partial loss of JAG1 protein due to mutations in one allele of the JAG1 gene is sufficient to disrupt normal bile duct development, resulting in the lack of bile ducts seen in ALGS (4). The type of mutation is variable and may be a point mutation, exon and whole gene deletion, or a microdeletion of 20p12 localized on the short arm of chromosome 20 (20p11.2–20p12) (12). It mainly includes protein truncation variants (insertion-deletions, nonsense, splice, full gene deletions, and partial gene deletions), which suggest a disease mechanism for reduced gene dosage leading to JAG1 haploinsufficiency (13). Biallelic variants in JAG1 have not been described and are likely to be lethal. In 1%–2% of affected patients, ALGS is caused by heterozygous pathogenic variants in NOTCH2 on chromosome 1p13.2, and no pathogenic variants were found in about 4% of affected patients, suggesting that some other genes may play a pathogenic role in this subgroup of cases (12). Impaired Notch2 signaling can lead to defects in cardiac embryogenesis. Mesodermal cells fail to differentiate into cardiomyocytes; endocardial cushioning defects and right heart valvular anomalies also may occur. Jagged1 is required for angiogenesis and is found to be strongly expressed in all major systemic arteries. Thus, its deficiency leads to the various vascular abnormalities observed in ALGS (7). Except for JAG1 and NOTCH2, no other genes other than Notch signaling genes were found to cause ALGS (14). Both cases that we reported were heterozygous variants in the JAG1 gene on chromosome 20, and no variants were detected in the NOTCH2 gene.

Cholestasis is initially treated with antipruritic medications, commonly including ursodeoxycholic acid, cholestyramine, rifampicin, ondansetron and naltrexone (13). In intractable cases, plasmapheresis has been shown to provide temporary relief from pruritus. Partial biliary diversion (external or internal) is indicated in patients with ALGS who present with isolated pruritus that has failed to respond to medication (4). Even this surgical technique does not alter the natural course of liver disease in ALGS. LT in ALGS is primarily used for end-stage liver disease leading to synthetic failure and/or portal hypertension. The median age of LT is 28 months, and the most common indication is intractable pruritus (7). > 40% of patients with ALGS receive a LT before the age of 5 years, and the estimated liver transplant-free survival rate is approximately 24% by age 18.5 years, suggesting the need for new treatment norms (15). Maralixibat and odevixibat block bile acid reabsorption in the intestine, lowering serum bile acids and relieving pruritus (16, 17). Therapies that address the root cause of the disease, i.e., reduction of JAG1-NOTCH2 signaling, are still needed to improve the overall treatment outcome of ALGS.

## Conclusions

We report two cases of infants with ALGS in which BA was ruled out by comprehensive examination, KPE was avoided, and the JAG1 mutation spectrum was expanded. Broadening the mutational spectrum may help geneticists to better identify etiologic mutations that cause ALGS.

## Data Availability

The datasets presented in this study can be found in online repositories. The names of the repository/repositories and accession number(s) can be found here: ncbi.nlm.nih.gov/, accession numbers: SRR21729814, SRR21729813.
